# A new inflammatory parameter can predict delayed intracranial hemorrhage following ventriculoperitoneal shunt

**DOI:** 10.1038/s41598-021-93315-4

**Published:** 2021-07-02

**Authors:** Shiwei Li, Hongcai Wang, Feng Li, Maosong Chen, Pandi Chen

**Affiliations:** 1 Neurosurgery Department of Ningbo Medical Center Lihuili Hospital, Zhejiang, China; 2 Medical Imaging Department of Ningbo Medical Center Lihuili Hospital, Zhejiang, China

**Keywords:** Hydrocephalus, Neuroimmunology

## Abstract

Delayed intracerebral hemorrhage (DICH) secondary to ventriculoperitoneal (VP) shunt is considered to be a potentially severe event. This study aimed to investigate the association between a ratio of postoperative neutrophil-to-lymphocyte ratio to preoperative neutrophil-to-lymphocyte ratio (NLRR) and DICH secondary to VP shunt. We performed a retrospective review of patients who underwent VP shunt between January 2016 and June 2020. Multivariable logistic regression analysis was used to assess the association of DICH and NLRR. Then patients were divided into two groups according to the optimal cut-off point of NLRR, propensity score matching (PSM) method was performed to reconfirm the result. A total of 130 patients were enrolled and DICH occurred in 29 patients. Elevated NLRR and history of craniotomy were independent risk factors for DICH secondary to VP shunt. The optimal cut off point of NLRR was 2.05, and the sensitivity was 89.7%, the specificity was 63.4%. Patients with NLRR > 2.05 had much higher incidence of DICH (40.6% vs 4.5%). Our finding suggested that DICH following VP shunt was not a rare complication and elevated NLRR could independently predict DICH. Inflammatory responses might play an important role in the development of DICH following VP shunt.

## Introduction

Ventriculoperitoneal (VP) shunt is the most common treatment for hydrocephalus^1^, and the placement of VP shunt is one of the routine neurosurgical procedures worldwide^2^. The common complications secondary to VP shunt are shunt obstruction, infection, seizure, subdural hemorrhage and shunt malfunction^3,4^. Mild hemorrhage is frequently observed in the ventricle or in the parenchyma soon after operation^5^, and the rate could be up to 43.1%^6^. However, the delayed intracerebral hemorrhage (DICH) is considered to be a rare but potentially severe event^7^, the mortality is as high as 50%^4^. The risk factors and underlying mechanisms of DICH are still not fully elucidated. There are several risk factors proposed by previously published articles^8–11^: (1) advanced age, age ≥ 60 years; (2) history of craniotomy; (3) delayed partial thromboplastin time (PTT); (4) brain edema around catheter; (5) postoperative manipulation of valve system; (6) postoperative anticoagulation.

A recent study pointed out that the systemic inflammatory responses might be involved in the pathologic process of active cerebral hemorrhage^12^. We hereby hypothesize that inflammatory response is one of the mechanisms associated with DICH following VP shunt. The neutrophil-to-lymphocyte ratio (NLR), as a rapid and economic biomarker of systemic inflammation^13^, has been a dependable predictor of clinical outcome in patients with spontaneous intracerebral hemorrhage^14^ and traumatic brain injury^15^. Considering that the value of NLR is greatly influenced by the basic systemic inflammatory statuses such as pneumonia or urinary infection, we proposed a new parameter named NLRR, what is a ratio of postoperative NLR to preoperative NLR. In this study, we sought to test the hypothesis that elevated NLRR is associated with the DICH secondary to VP shunt.

## Methods

### Patient selection

We performed a retrospective review of patients who underwent VP shunt between January 2016 and June 2020 from the Neurosurgery Department of Ningbo Medical Center Lihuili Hospital. Inclusion criteria were as follows: (1) age ≥ 18 years; (2) the diagnosis of hydrocephalus was confirmed by clinical symptoms and imaging examination, and VP shunt was performed in our hospital; (3) laboratory tests (blood routine and coagulation function) within 5 days before VP shunt, and blood routine on the first morning after VP shunt; (4) postoperative brain computed tomography (CT) scan was performed on the first day after operation (later than the postoperative test of blood routine), and at least one CT scan was performed within 5–10 days after operation. The exclusion criteria were as follows: (1) patients on a regimen of anticoagulant or antiplatelet therapy; (2) patients with Ommaya reservoir implantation, the Ommaya tube was directly connected to the shunt pump without ventricular puncture, or the Ommaya tube was removed during the surgery; (3) cranioplasty and VP shunt were performed simultaneously; (4) a revision of the VP shunt; (5) early intracerebral hemorrhage after VP shunt, which was defined as bleeding on the first day after operation.

**Figure 1:**
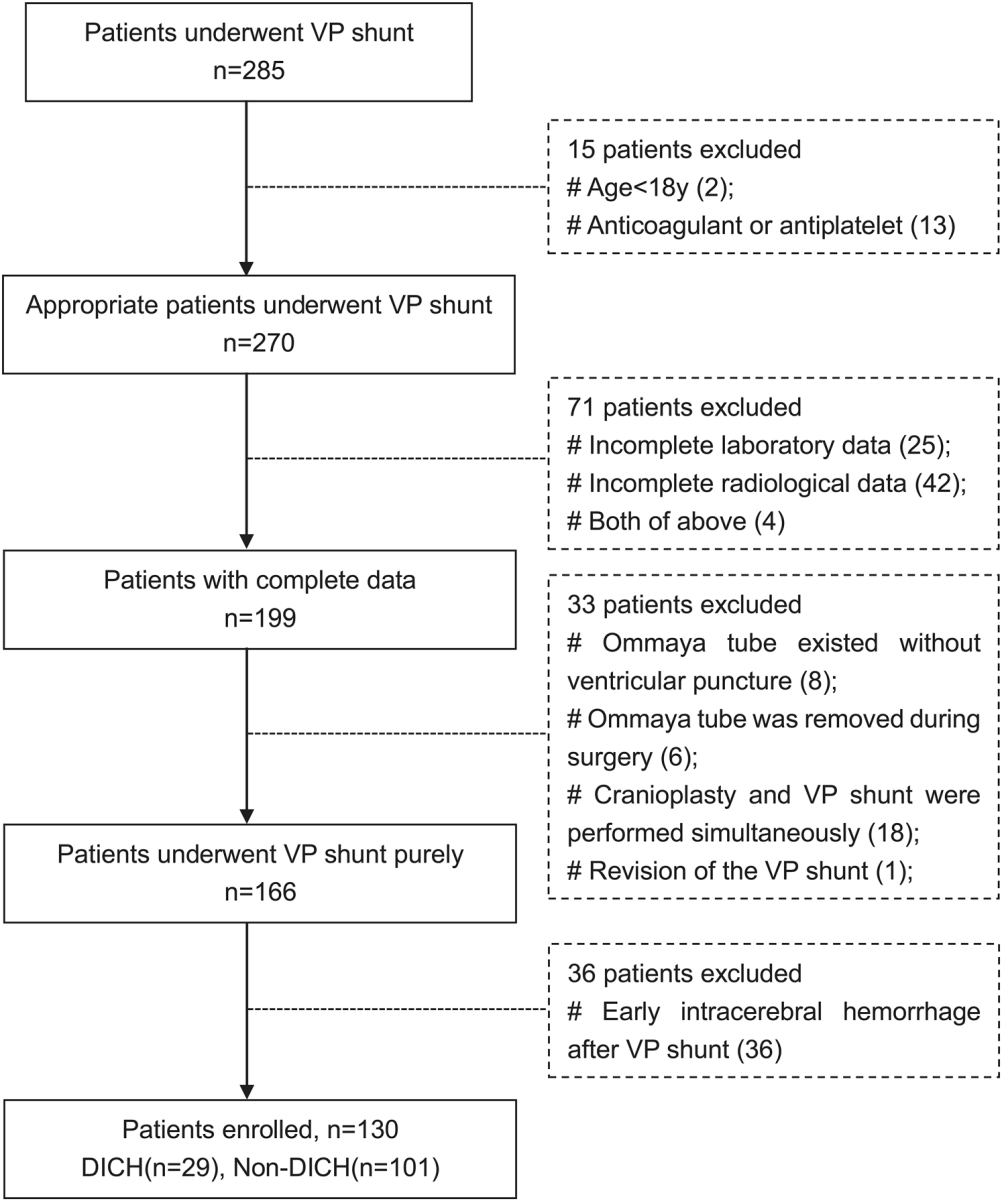
Flowchart of patient selection.

DICH was defined as subsequent hemorrhage in the ventricle or the parenchyma along the catheter path which was not found in the CT scan on the first day after operation. The patients enrolled in the study were divided into two groups according to whether or not DICH. The patient flowchart was summarized in Fig. 1.

### Data collection

Demographic characteristics and clinical variables were collected such as: sex and age; history of hypertension and diabetes; history of craniotomy and skull defect; preoperative pneumonia and Glasgow Coma Scale (GCS); primary intracranial lesions including normal hydrocephalus, trauma, spontaneous intracerebral hemorrhage(ICH), tumor and inflammation; hydrocephalus types including low pressure hydrocephalus (LPH, cerebrospinal fluid pressure < 80 mm H_2_O), normal pressure hydrocephalus (NPH, 80 mm H_2_O ≤ cerebrospinal fluid pressure ≤ 180 mm H_2_O), high pressure hydrocephalus (HPH, cerebrospinal fluid pressure > 180 mm H_2_O). For the patients with DICH secondary to VP Shunt, the onset day of hemorrhage, types of hemorrhage, with or without symptom after hemorrhage and the Glasgow outcome scale (GOS) were also collected. Laboratory variables were retrieved from our hospital’s database. International normalized ration (INR), prothrombin time (PT), activated partial thromboplastin time (APTT), serum thrombocytes, neutrophils and lymphocytes were collected within 5 days before operation. Postoperative serum neutrophils and lymphocytes were collected on the first morning after operation. All of the preoperative and postoperative brain CT scans were obtained, and respectively reviewed by one neurosurgeon and one radiologist who blinded to the detail information (demographic and clinical variables, laboratory data) of patients. The different opinions between them were resolved by consultation. The volume of hematoma was calculated by 3D slicer (version 4.10.2). Besides, postoperative cerebral edema around catheter on the first postoperative CT scan was also collected.

### Procedural technique

All of the patients were implanted with Medtronic Strata Adjustable Pressure Valve Systems, and the initial pressures were collected. The standard technique for VP shunt was employed, and the catheter was placed into the left or right anterior frontal horn via a bur hole at Kocher’s point. The postoperative manipulation of valve system was recorded before the occurrence of DICH, or within 15 days after operation of patient without DICH.

### Statistical analysis

SPSS version 26.0 (IBM Corporation, Armonk, New York, USA) and R Software (version 4.0.2) were used for data analysis with statistical significance was defined as P < 0.05. The Kolmogorov–Smirnov test was used to determine the distributions of continuous variables. The continuous variables with non-normally distributions were analyzed by Mann–Whitney test, presented as the median (50th) with interquartile range (IQR). Categorical variables were presented as number (proportion). Mann–Whitney test was used for ordered categorical variables, and unordered categorical variables were analyzed by Pearsons chi-square test, Continuous correction chi-square test or Fishers exact test. The variables with P < 0.1 and variables proposed by previously published articles, were selected by Least Absolute Shrinkage and Selection Operator (LASSO) regression, and then included into multivariable logistic regression analysis to assess the association of DICH and NLRR. The predictive value of NLRR for DICH following VP shunt in patients was evaluated by receiver operating characteristic (ROC) curve analysis. According to the optimal cut-off point of NLRR (the point at which the value of “sensitivity + specificity—1” was maximum), patients were divided into two groups (NLRR ≤ cut-off point group and NLRR > cut-off point group). Propensity score matching (PSM) method was performed to adjust for imbalances of patients’ characteristics between two groups. Covariates such as sex, age, history of hypertension and diabetes, history of craniotomy and skull defect, preoperative pneumonia and GCS grade, primary intracranial lesion, hydrocephalus type, preoperative PT and APTT, preoperative INR and PLT, puncture site, initial pressure of vale system, brain edema around catheter and postoperative manipulation of valve system were matched at a ratio of 1:1 using a caliper width of 0.2. The estimation algorithm of propensity score was logistic regression and matching algorithm was nearest neighbor matching. After PSM, 82 patients (NLRR ≤ 2.05 group: n = 41, NLRR > 2.05 group: n = 41) were selected to analysis.

### Ethics approval and consent to participate

This study was performed in accordance with the Declaration of Helsinki of the World Medical Association, and all procedures were carried out in accordance with the approved guidelines and regulations. This study was approved by our institutional ethics committee (Ningbo Medical Center Lihuili Hospital, Approval Number: KY2020PJ111). Participant data were retrospectively reviewed and deidentified. Because of anonymization, consent was waived by the ethics committee of Ningbo Medical Center Lihuili Hospital.

## Results

### Characteristics of the patients

A total of 130 patients underwent VP shunt were included in this study. Because there were 36 patients with early intracerebral hemorrhage after VP shunt who were excluded, the overall incidence of DICH secondary to VP shunt was 17.5% (29/166). The median age of the 130 patients was 60 years, and 78 (60.0%) patients were male (Table 1). The 130 patients were divided into two groups: non-DICH group (n = 101) and DICH group (n = 29). Characteristics and clinical data were compared between the two groups and shown in Supplementary Table S1 online. Most of the data were comparable, except history of craniotomy and preoperative NLR, postoperative NLR, NLRR. 15 patients in the DICH group (51.9%) presented a history of craniotomy, while the number in the non-DICH group is 34 (33.7%) (P = 0.008). Lower preoperative NLR (P = 0.037) and higher postoperative NLR (P = 0.001) were observed in patients with DICH secondary to VP shunt. NLRR in the DICH group was much higher than that in the non-DICH group (P < 0.001).

**Table 1:** Characteristics and clinical data of the patients.

Variables	n (%) or median [IQR]
**Demographics**	
Male sex	78 (60.0)
Age (years)	60.0 [53.0–66.0]
**Clinical history**	
Hypertension	49 (37.7)
Diabetes mellitus	16 (12.3)
Craniotomy	75 (57.7)
Skull defect	34 (26.2)
Preoperative pneumonia	44 (33.8)
Preoperative GCS	13 [9–15]
**Primary intracranial lesion**	
Normal hydrocephalus	8 (6.2)
Trauma	56 (43.1)
ICH	48 (36.9)
Tumor	15 (11.5)
Inflammation	3 (2.3)
**Hydrocephalus type**	
LPH	11 (8.5)
NPH	101 (77.7)
HPH	18 (13.8)
**Laboratory test**	
Pre-PT (s)	11.9 [11.3–12.6]
Pre-APTT (s)	30.2 [28.0–32.2]
Pre-INR	1.04 [1.00–1.09]
Pre-PLT (*10^3^/μL)	216 [173–264]
Pre-NLR	2.67 [1.60–4.3.8]
Post-NLR	5.45 [3.59–7.35]
NLRR	2.02 [1.38–3.16]
**Puncture site**	
Left precornu	45 (34.6)
Right precornu	85 (65.4)
**Initial pressure of vale system**	
1.0	30 (23.1)
1.5	67 (51.5)
2.0	28 (21.5)
2.5	5 (3.8)
Brain edema around catheter	26 (20.0)
Manipulation of valve system	54 (41.5)

*GCS* Glasgow Coma Scale, *ICH* spontaneous intracerebral hemorrhage, *LPH* low pressure hydrocephalus, *NPH* normal pressure hydrocephalus, *HPH* high pressure hydrocephalus, *Pre-PT* preoperative prothrombin time, *Pre-APTT* preoperative activated partial thromboplastin time, *Pre-INR* preoperative international normalized ration, *Pre-PLT* preoperative serum thrombocyte, *Pre-NLR* preoperative neutrophil-to-lymphocyte ratio, *Post-NLR* postoperative neutrophil-to-lymphocyte ratio, *NLRR* a ratio of post-NLR to pre-NLR.

### Association between elevated NLRR and DICH

Hypertension, history of craniotomy, preoperative APTT, preoperative PTL, preoperative NLR, postoperative NLR, NLRR (P < 0.1) and age, brain edema around catheter, postoperative manipulation of valve system (proposed by previously published articles), were included. The linear relationships between the continuous independent variables and the logit conversation of dependent variable were confirmed by Box–Tidwell test. The indicators of multicollinearity (tolerance, variance inflation factor, eigenvalue, condition index) were statistically tested, and the results of eigenvalue and condition index showed that there was multicollinearity among above variables. In order to solve the multicollinearity problem, Least Absolute Shrinkage and Selection Operator (LASSO) analysis in R software (package glmnet) was used to select variables (Supplementary Fig. S1 online). Finally, five variables including hypertension, history of craniotomy, postoperative NLR, NLRR, postoperative manipulation of valve system were selected into the multivariate logistic regression model. After adjustment of potential confounding variables, NLRR was considered as an independent risk factors for DICH, as well as history of craniotomy (Table 2).

**Table 2:** Association between elevated NLRR and DICH secondary to VP shunt.

	Crude		Adjusted	
	OR (95% CI)	P	OR (95% CI)	P
Hypertension	2.111 (0.914–4.877)	0.080	1.742 (0.612–4.962)	0.298
Craniotomy	3.612 (1.356–9.620)	0.010	3.394 (1.060–10.869)	0.040
Post-NLR	1.142 (1.025–1.274)	0.016	1.110 (0.971–1.267)	0.125
NLRR	2.839 (1.843–4.374)	< 0.001	2.792 (1.747–4.460)	< 0.001
Manipulation of valve system	0.682 (0.288–1.613)	0.383	0.414 (0.134–1.275)	0.124

*OR* odds ratio, *CI* confidence interval, *pre-NLR* preoperative neutrophil-to-lymphocyte ratio, *Post-NLR* postoperative neutrophil-to-lymphocyte ratio, *NLRR* a ratio of post-NLR to pre-NLR.

### Receiver operating characteristic curve analysis

**Figure 2:**
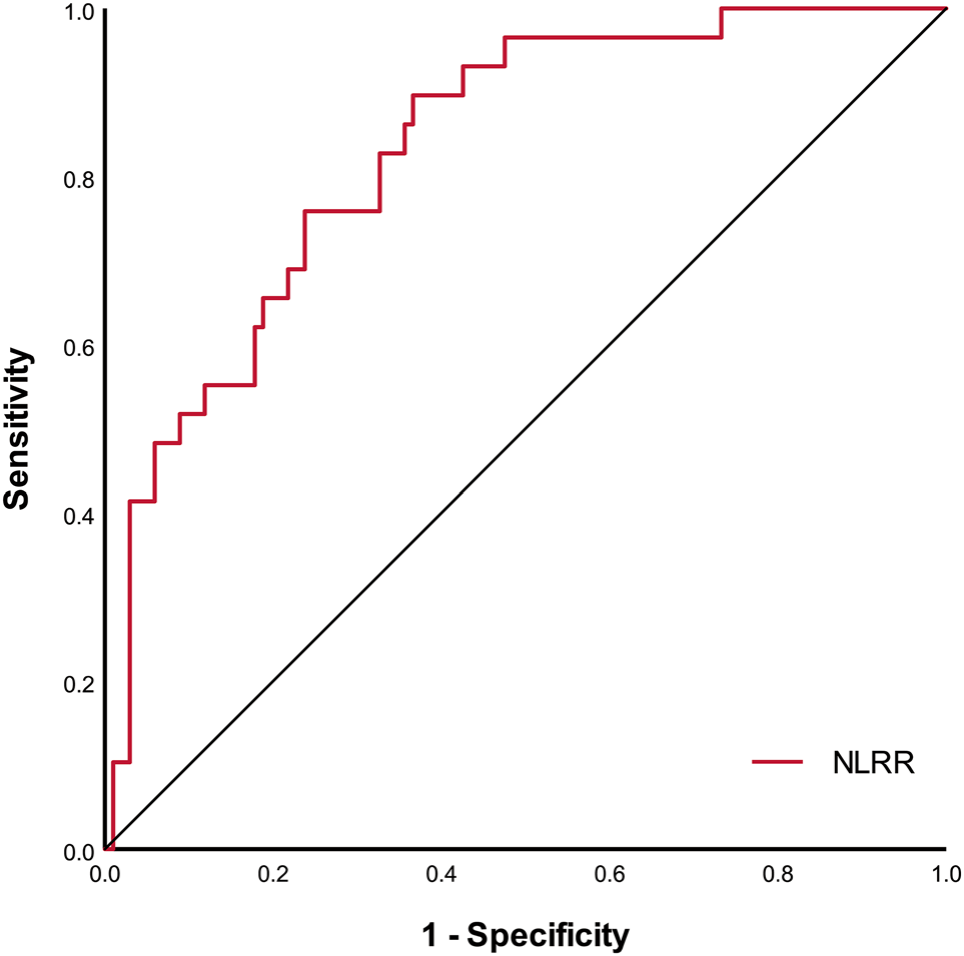
Receiver operating characteristic curves of NLRR to predict DICH. Area under the curve was 0.832 (95% CI 0.754–0.910; P < 0.001) for NLRR.

ROC analysis of NLRR regarding DICH was shown in Fig. 2, area under the curve (AUC) was 0.832, with a 95% CI 0.754–0.910 (P < 0.001). The optimal cut off point of NLRR as a predictor for DICH was determined as 2.05, and the sensitivity was 89.7%, the specificity was 63.4%, the positive predictive value was 40.6%, the negative predictive value was 95.5%.

### Propensity score matching (PSM) analysis

**Figure 3:**
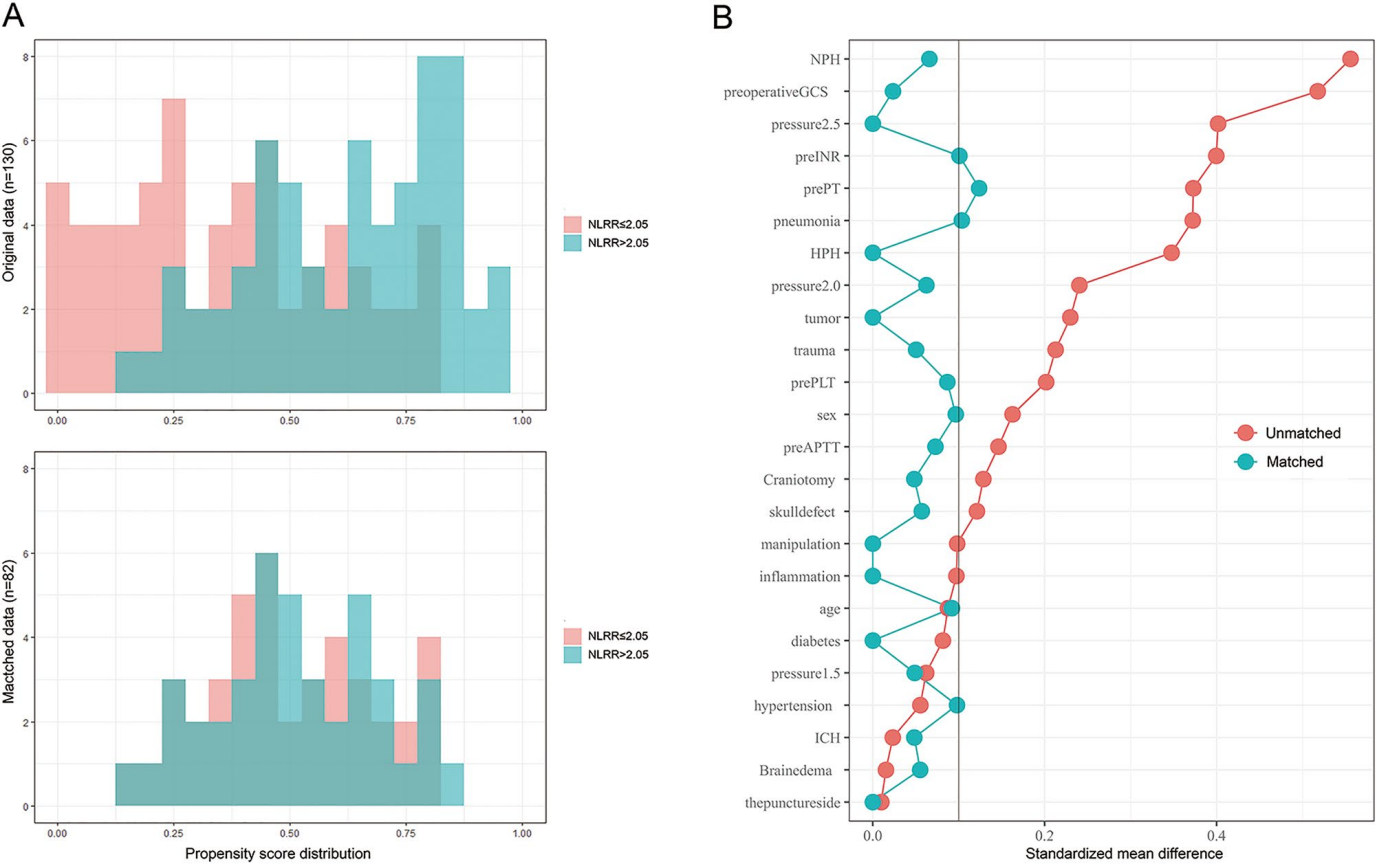
Propensity score distributions (**A**) and standardized mean differences (**B**) after PSM. *GCS* Glasgow Coma Scale, *ICH* spontaneous intracerebral hemorrhage, *NPH* normal pressure hydrocephalus, *HPH* high pressure hydrocephalus, *Pre-PT* preoperative prothrombin time, *Pre-APTT* preoperative activated partial thromboplastin time, *Pre-INR* preoperative international normalized ration, *Pre-PLT* preoperative serum thrombocyte.

Considering that there were five independent variables included in the multivariate logistic regression analysis, the model might be unstable. PSM analysis method^16^ was conducted to confirm the results. After PSM, the propensity score distributions were similar between the two groups (NLRR ≤ 2.05 group and NLRR > 2.05 group) (Fig. 3A), and the standardized mean differences were much smaller than before (Fig. 3B). The covariates were generally balanced between two groups. Univariable logistic regression analysis showed that the incidence of DICH following VP shunt was much higher in NLRR > 2.05 group than NLRR ≤ 2.05 group (p = 0.025), and the odds ratio was 11.25 (95% CI 1.35–93.50). It was similar to the result obtained from the adjusted multivariable logistic regression analysis before PSM, which was 10.01 (95% CI 1.64–61.25; p = 0.013) (Table 3).

**Table 3:** Association between NLRR(> 2.05 vs ≤ 2.05) and DICH secondary to VP shunt.

	Original data (n = 130)*		After PSM (n = 82)**	
	OR (95% CI)	P	OR (95% CI)	P
NLRR (> 2.05 vs ≤ 2.5)	10.01 (1.64–61.25)	0.013	11.25 (1.35–93.50)	0.025

*PSM* propensity score matching, *OR* odds ratio, *CI* 95% confidence interval, *NLRR* a ratio of post-NLR to pre-NLR.

*Multivariable logistic regression analysis adjusted by age, hypertension, preoperative APTT, preoperative PLT, brain edema around catheter, postoperative valve manipulation, preoperative NLR, postoperative NLR, history of craniotomy.

**Univariable logistic regression analysis.

### Characteristics of hemorrhage in the patients with DICH

**Figure 4:**
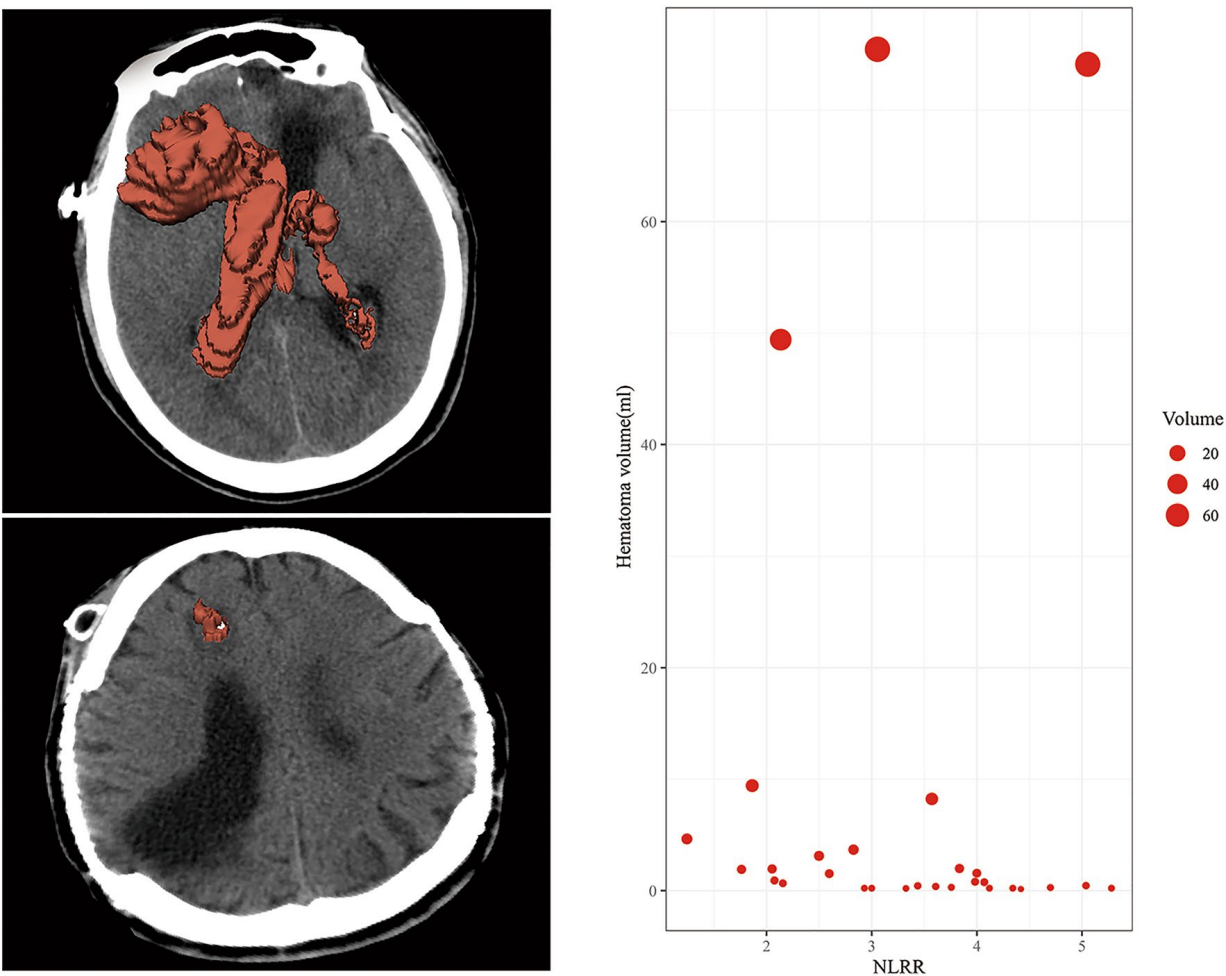
The scatter plot of NLRR value and hematoma volume.

The mean onset day of DICH after operation was 6.10 ± 0.53 day, ranged from 2 to 13 day. Intraventricular hemorrhage was the most common type, which was presented in 13 patients (44.8%). 16 patients (55.2%) with hematoma volume less than 1 ml, only three patients (10.3%) had hematoma volume more than 15 ml, and the maximum hematoma volume was 74.5 ml. 6 patients (20.7%) were found to be symptomatic, such as vomiting, epilepsy and decreased consciousness. 15 patients (51.7%) had a GOS = 3 at the time of discharge, while only one patient (3.4%) had a GOS = 1, whose hematoma volume was 74.1 ml. The scatter plot of NLRR and hematoma volume was shown in Fig. 4, it seemed that there was no correlation between the NLRR and hematoma volume.

## Discussion

DICH is one of complications of VP shunt surgery and was first reported in 1985^17^. It was considered to be a rare complication with incidence varies from 0.4 to 4%^4,5,8,9,18^. However, the incidence might be underestimated. Patient with small hematoma might be missed if CT scan was not performed frequently^4^. An article published in 2017 reported that the incidence of postoperative DICH was 7.8% (17/218)^10^. In our study, the incidence was 17.5%, which was much higher than previous studies. Another article published in 2018 reported that the incidence was 23.7% (34/143) without excluding patients with anticoagulant and antiplatelet therapy^11^. Just as our study, they included the patients with hematoma volume less than 1 ml which would be ignored easily and maybe it is the reason. The incidence of symptomatic DICH was 3.6% (6/166) in our study.

The mechanisms underlying the DICH secondary to VP shunt are still controversial. Several hypotheses have been proposed: (1) erosion of cerebral vasculature by the insertion of catheter; (2) fragility of cerebral tissue caused by advanced age, craniotomy, trauma or stroke; (3) disseminated intravascular coagulation (DIC) induced by VP shunt; (4) coagulopathy, anticoagulant or antiplatelet therapy; (5) sudden change of intracranial pressure after manipulation of the valve system^7,19^. Savitz and Bobroff^5^ pointed out that the mechanism of DICH was more likely erosion of surface or deeper small vessel by catheter. This opinion was supported by most reports because most hematomas located along the catheters^4^. The hypercapnia, hypoxia and venous congestion might encourage developing of hematoma at the sites of injury just as the mechanism of traumatic delayed ICH^20^. A study published in 2017 found that postoperative cerebral edema around the catheter observed on the first CT scan was an independent risk factor for DICH^9^, which might be a sign of vascular erosion. Nevertheless, this difference was not found in our study. Advanced age, history of craniotomy were considered to be the risk factors for DICH secondary to VP shunt in several articles^8–11^, and these factors might increase the fragility of cerebral tissue. However, one article published in 2017 reported that there was no difference between two groups with respect to age^10^. In our study, we also found that history of craniotomy was an independent risk factor for DICH, while advanced age was not. DIC induced by catheter insertion was considered to be another potential mechanism of DICH. Two cases of DIC associated with VP shunt were reported^21,22^. A Korean study found that prolonged partial thromboplastin time was major risk factor of DICH^11^. Some studies showed that dual antiplatelet therapy and postoperative anticoagulation therapy would increase the risk of DICH^10,23^. As for our study, we excluded the patients with anticoagulant or antiplatelet therapy in order to control the confounding factors, and we found that preoperative PT, APTT, INR and PLT were not risk factors for DICH. Two articles presented that postoperative manipulation of valve system might be a risk factor for DICH secondary to VP shunt^4,8^, which was not supported in our study either.

As the main purpose of the study, we concluded that elevated NLRR could independently predict DICH secondary to VP shunt, which suggested that inflammatory responses might play an important role in the development of DICH. Catheters of VP shunt are made of silicones and may not be immunologically inert^24^. A study pointed out that immune response might be elicited by VP shunt in some patients and could lead to shunt malfunctions^25^. Neutrophils and giant cells were found on the surface of catheters by scanning electron microscopy^26^. In view of the above, we hypothesize that the inflammatory responses may arise from the stimulation of catheter as a foreign body. The acute inflammation phase of foreign body reaction against biomaterials is characterized by migration, adhesion, activation of neutrophils and mast cells, and lasts for hours to few days^27^. Just as the process of brain injury^28^, neutrophils’ number increase greatly in the peripheral blood and they can enter central nervous system through the damaged blood brain barrier early^15^. Recruitment and infiltration of neutrophils around catheter could induce neurotoxicity by following pathways: production of cytotoxic mediators and proinflammatory cytokines, activation of matrix metalloproteinases and increase of oxidate stress^29–31^. The ensuing further destruction of blood brain barrier, cellular swelling and increased permeability of capillary might trigger cerebral edema and active bleeding^32–36^. Lymphocytes play an important part in the cellular and humoral immune. It was found that autoreactive T cells could promote vascular reconstruction and healing after cerebral trauma^37^. Studies showed that depletion of blood neutrophils would reduce Blood–Brain Barrier breakdown^38,39^, while increase of blood regulatory T lymphocytes could alleviate the degradation of Blood–Brain Barrier^40^. Therefore, Elevated NLRR (an increase of blood neutrophils or decrease of lymphocytes) might aggravate the degradation of Blood–Brain Barrier and induce DICH secondary to VP shunt. We suggest that the patients with NLRR > 2.05 should be more carefully observed after VP shunt. However, there was no correlation between the NLRR and hematoma volume. The volume of hematoma might be affected by many factors, such as blood pressure, coagulation function and so on. Since limited understanding of the mechanisms of DICH, our finding would also contribute to identify potential preventive and curative strategies.

NLRR, as a new inflammatory parameter, has smaller variation range than NLR, and can roughly represent the change of inflammatory status due to surgery (including anesthesia) and perioperative treatment. Maybe it could be used as predictors of other diseases requiring surgery, such as postoperative rebleeding of ICH following minimally invasive surgery.

There are several limitations in our study. The first, it is a retrospective study with small sample size, and a quarter of patients were excluded due to incomplete laboratory or radiological data, which may induce potential selection of bias. The second, there were five variables included in the multivariate logistic regression analysis, the model might be unstable, even though the result was reconfirmed by PSM analysis. The third, the time of ventricular puncture attempt and postoperative treatments such as hemostatic therapy were not included in our study, which might be confounding factors. Finally, preoperative neutrophils and lymphocytes were collected within 5 days before surgery. In general, patient’s condition was stable before operation, fever or other unusual situation would lead to cancellation of operation, and the laboratory indexes would not change greatly in these days. However, there were still small deviations in these data and they could not represent the preoperative inflammatory status accurately. NLRR’s predictive value should be verified by further larger prospective studies.

## Conclusions

In this study, we proposed a new parameter named NLRR, and suggested that DICH following VP shunt was not a rare complication. History of craniotomy and elevated NLRR were independent risk factors for DICH secondary to VP shunt. According to the results, we proposed that inflammatory responses might play an important role in the developing of DICH. More attention should be paid to the patients with NLRR > 2.05 after VP shunt.

## Supplementary Information

Supplementary Figure S1.

Supplementary Table S1.

## Data Availability

All data are available within the text of the article. Further anonymized data could be made available to qualified investigators upon reasonable request.

## Abbreviations

DICH: Delayed intracerebral hemorrhage
VP: Ventriculoperitoneal
NLRR: A ratio of postoperative neutrophil-to-lymphocyte ratio to preoperative neutrophil-to-lymphocyte ratio
PSM: Propensity score matching
OR: Odds ratio
CI: Confidence interval
ROC: Receiver operating characteristic
AUC: Area under the curve
NLR: Neutrophil-to-lymphocyte ratio
GCS: Glasgow Coma Scale
GOS: Glasgow outcome scale
ICH: Spontaneous intracerebral hemorrhage
IQR: Interquartile range
INR: International normalized ration
PT: Prothrombin time
APTT: Activated partial thromboplastin time
PLT: Thrombocytes
