# Supplementary figures and images for: A new inflammatory parameter can predict delayed intracranial hemorrhage following ventriculoperitoneal shunt
Source: Sci Rep. 2021 Jul 2;11:13763. doi: 10.1038/s41598-021-93315-4 (PMC8253783; doi:10.1038/s41598-021-93315-4)

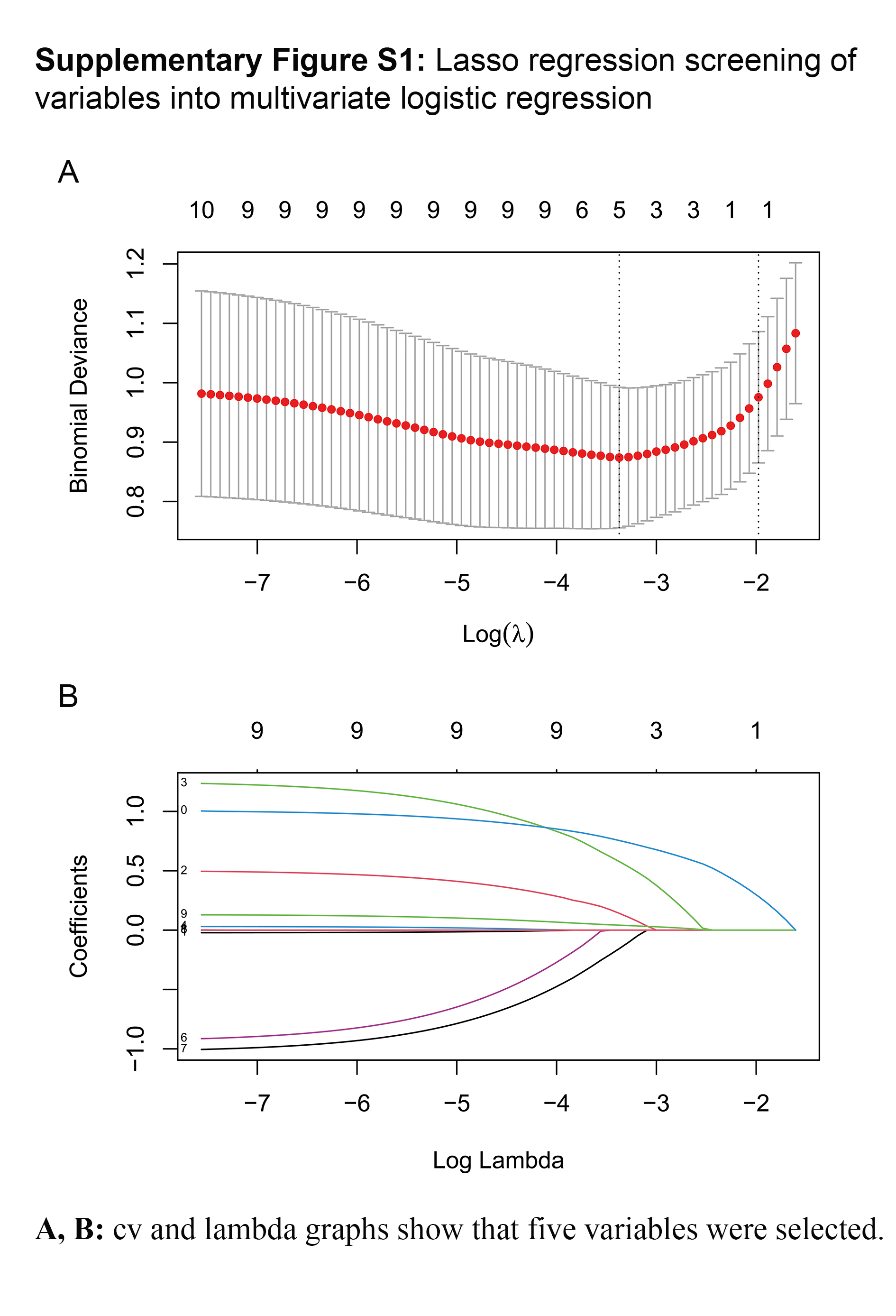

Supplement: Supplementary file 1 — Supplementary Figure S1. [file 41598_2021_93315_MOESM1_ESM.tif]
