# Supplementary material for: A new inflammatory parameter can predict delayed intracranial hemorrhage following ventriculoperitoneal shunt
Source: Sci Rep. 2021 Jul 2;11:13763. doi: 10.1038/s41598-021-93315-4 (PMC8253783; doi:10.1038/s41598-021-93315-4)
Supplement: Supplementary file 2 — Supplementary Table S1. [file 41598_2021_93315_MOESM2_ESM.docx]

**Supplementary Table S1** The comparation of Non-DICH group and DICH group

| **Variables** | **Non-DICH (n=101)** | **DICH (n=29)** | **P** |
| --- | --- | --- | --- |
| **Demographics** |  |  |  |
| **Male sex, n(%)^▲^** | 59(58.4) | 19(65.5) | 0.491 |
| **Age (y), median[IQR] ^■^** | 60.0[54.0-67.5] | 60.0[50.5-65.5] | 0.667 |
| **Clinical history, n(%)** |  |  |  |
| **Hypertension^▲^** | 34(33.7) | 15(51.9) | 0.077* |
| **Diabetes mellitus^▼^** | 13(12.9) | 3(10.3) | 0.965 |
| **Craniotomy^▲^** | 52(51.5) | 23(79.3) | 0.008** |
| **Skull defect^▲^** | 24(24.8) | 9(31.0) | 0.497 |
| **Preoperative pneumonia^▲^** | 33(32.7) | 11(37.9) | 0.598 |
| **Preoperative GCS, median[IQR]^■^** | 12[9-15] | 13[9-15] | 0.674 |
| **Primary intracranial lesion, n(%)^◆^** |  |  | 0.243 |
| **normal hydrocephalus** | 7(6.9) | 1(3.4) |  |
| **trauma** | 39(38.6) | 17(58.6) |  |
| **ICH** | 39(38.6) | 9(31.0) |  |
| **tumor** | 14(13.9) | 1(3.4) |  |
| **Inflammation** | 2(2.0) | 1(3.4) |  |
| **Hydrocephalus type, n(%)^■^** |  |  | 0.236 |
| **LPH** | 9(8.9) | 2(6.9) |  |
| **NPH** | 75(74.3) | 26(89.7) |  |
| **HPH** | 17(16.8) | 1(3.4) |  |
| **Laboratory test, median[IQR]^■^** |  |  |  |
| **Pre-PT(seconds)** | 11.9[11.4-12.6] | 11.8[11.2-12.5] | 0.503 |
| **Pre-APTT(seconds)** | 29.7[27.8-32.1] | 31.2[29.5-32.5] | 0.073* |
| **Pre-INR** | 1.04[1.00-1.10] | 1.04[0.96-1.09] | 0.544 |
| **Pre-PLT(*10^3^/μL)** | 224[176-269] | 190[157-251] | 0.084* |
| **Pre-NLR** | 2.89[1.78-4.61] | 2.27[1.50-2.85] | 0.037** |
| **Post-NLR** | 4.75[3.27-7.04] | 6.08[5.46-9.25] | 0.001** |
| **NLRR** | 1.69[1.15-2.44] | 3.44[2.33-4.10] | <0.001** |
| **Puncture site, n(%)^▲^** |  |  | 0.385 |
| **Left precornu** | 33(32.7) | 12(41.4) |  |
| **Right precornu** | 68(67.3) | 17(58.6) |  |
| **Initial pressure of vale system, n(%)^■^** |  |  | 0.571 |
| **1.0** | 24(23.8) | 6(20.7) |  |
| **1.5** | 53(52.5) | 14(48.3) |  |
| **2.0** | 19(18.8) | 9(31.0) |  |
| **2.5** | 5(5.0) | 0(0.0) |  |
| **Brain edema around catheter, n(%)^▲^** | 21(20.8) | 5(17.2) | 0.674 |
| **Manipulation of valve system, n(%)^▲^** | 44(43.6) | 10(34.5) | 0.382 |

Abbreviations: GCS, Glasgow Coma Scale; ICH, spontaneous intracerebral hemorrhage; LPH, low pressure hydrocephalus; NPH, normal pressure hydrocephalus; HPH, high pressure hydrocephalus; Pre-PT, preoperative prothrombin time; Pre-APTT, preoperative activated partial thromboplastin time; Pre-INR, preoperative international normalized ration; Pre-PLT, preoperative serum thrombocyte; Pre-NLR, preoperative neutrophil-to-lymphocyte ratio; Post-NLR, postoperative neutrophil-to-lymphocyte ratio; NLRR, a ratio of post-NLR to pre-NLR.

^■^Mann-Whitney U test; ^▲^Pearsons chi-square test; ^▼^Continuous correction chi-square test; ^◆^Fishers exact test

*P<0.1, **P<0.05
